# Hyperthermic Core‐Shell Silver‐Gold Nanoparticles: Green Synthesis and Adsorption‐Uptake by Macrophages, Fibroblasts and Cancer Cells

**DOI:** 10.1002/open.202400459

**Published:** 2025-02-19

**Authors:** E. Valdivieso, M. Zabala, A. Muñoz Noval, R. López‐Méndez, N. Carmona, A. Espinosa, F. J. García García, K. Boulahya, J. A. Lucas, L. Biancotto, U. Amador, M. T. Azcondo, C. Hurtado‐Marcos

**Affiliations:** ^1^ Pharmaceutical and Health Sciences Department Faculty of Pharmacy Universidad San Pablo-CEU CEU-Universities 28668 Boadilla del Monte Spain; ^2^ Universidad San Pablo-CEU CEU Universities Facultad de Farmacia Departamento de Química y Bioquímica Urbanización Montepríncipe, Boadilla del Monte E-28668 Madrid Spain; ^3^ Departamento de Física de Materiales Facultad de Físicas Universidad Complutense E-28040 Madrid Spain; ^4^ IMDEA Nanociencia c/ Faraday, 9 Madrid 28049 Spain; ^5^ Instituto de Ciencia de Materiales de Madrid Consejo Superior de Investigaciones Científicas calle Sor Juana Inés de la Cruz 3 28049- Madrid Spain; ^6^ ICTS-Centro Nacional de Microscopía Electrónica F. CC. Químicas, UCM Av. Complutense S/N 28040- Madrid Spain; ^7^ Departamento de Química Inorgánica I Facultad de Ciencias Químicas Universidad Complutense 28040 Madrid Spain

**Keywords:** Nanoparticles, Silver, Gold, Green synthesis, Plasmonic systems, Macrophages, Fibroblast, Cancer cells

## Abstract

Gold‐coated silver nanoparticles (Ag@AuNPs) are synthesized by green synthesis using *Vaccinium corymbosum* as reducing agent. The obtained Ag@AuNPs present a core‐shell structure with nanostar shape. The absorption spectrum of these nanoparticles shows a prominent band centred at 680 nm, within the optimal range for photothermal applications. Dispersions of Ag@AuNPs in water, 1.87 10^10^ NPs/mL, reach a temperature of 44.3 °C under laser excitation in 10 minutes, which is suitable for hyperthermia therapy. The internalization of Ag@AuNPs, at a concentration of 3 10^8^ NPs/ml, by macrophages (Raw 264.7), human fibroblasts (Hs27), and cancer cells (4T1) is confirmed by transmission electron microscopy. Cytotoxicity studies demonstrate that at this concentration the cells are viable.

## Graphical abstract

Promising silver star‐shaped nanoparticles coated with gold are synthesized in a green manner using *V. Corimbosum* as reducing agent. Structural and morphological characterization of the nanoparticles is performed, and their photothermal properties are studied. The viability of internalization and cytotoxicity of these nanoparticles in macrophages, fibroblasts, and T4 cells is also examined.

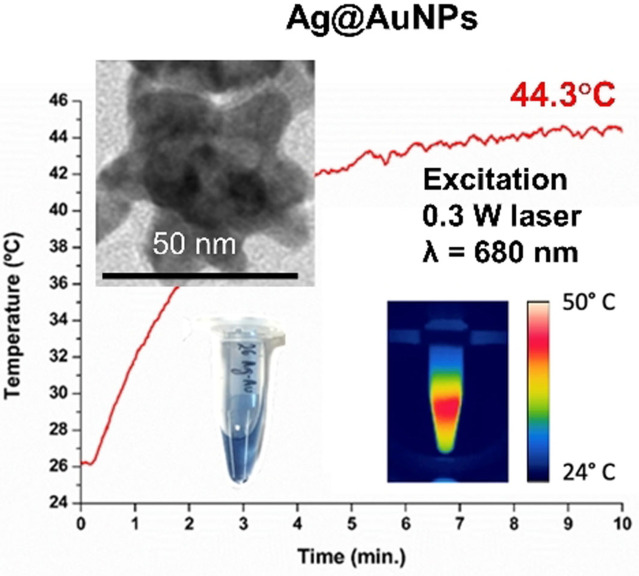

## 1 Introduction

The recently coined term “Thermoplasmonics” refers to the generation and manipulation of nanoscale heating associated with noble metal nanoparticles (NPs).^[1]^ Gold and silver NPs, which exhibit a light absorption band in the visible region, are the archetypal plasmonic systems for generating heat through a nanoscale confined light‐triggered effect. The plasmonic response of NPs depends on both intrinsic and extrinsic parameters of the nanomaterial itself.^[2]^ Despite more intense plasmonic absorption and greater scattering efficiency,^[3]^ silver NPs are scarcely used for hyperthermia applications because the surface of silver nanoparticles is chemically unstable.^[4]^ Particularly in physiological conditions, silver tends to oxidize or form nitrides that eventually dampen or extinguish the plasmon. In thermoplasmonics, these NPs have been mainly used as a therapeutic tool for cancer treatment as light‐to‐heat conversion (photoexcited hyperthermia) agents.[^3^, ^5^] This photothermal effect allows for the selective destruction of malignant cells or pathogens via controlled hyperthermia, minimizing damage to surrounding tissues.

In particular, gold nanostars, with their irregular surfaces and multiple spikes, exhibit enhanced efficiency in light absorption and scattering, amplifying their photothermal effect and making them especially useful for therapeutic and diagnostic applications.^[6]^ The ability to functionalize these nanostars with specific ligands also enables greater selectivity and efficiency in targeted therapy delivery, underscoring their importance in the development of personalized treatments and the fight against complex diseases.

Additionally, these could also be used in cancer diagnostics, imaging, biosensing, and gene and drug delivery.^[7]^ However, the potential toxicity of silver in the human metabolism, in chronic doses, can cause argyria syndrome or its toxicity to a large number of species in aquatic environments.^[8]^ Therefore, coating with a biocompatible gold shell confers chemical stability and improves biocompatibility, limiting its toxicity in the short and medium term. Thus, Ag core‐Au shell nanoparticle systems are highly interesting.^[9]^

These core‐shell nanoparticles have potential therapeutic importance; thus, it is essential to find environmentally friendly, effective, and economical synthesis methods. In this connection, green synthesis routes are one of the current trends since they fulfil all those aspects. These kinds of methods use natural sources such as microorganisms, fungi, yeasts, bacteria, plants, and plant extracts.^[10]^ In particular, plants stand as an outstanding resource for nanoparticle production. Besides their abundance, accessibility, ease of handling, and capacity to produce a variety of metabolites, many plants contain pharmacological components acting as coating and reducing agents during the nanoparticle synthesis process.^[10b]^ Terpenoids, flavonoids, ketones, aldehydes, amides, and carboxylic acids are among the plant compounds that directly contribute to the reduction of metal ions to form nanoparticles. Among them, phenolic compounds have been reported to be essential for the reduction of silver and other metals to form nanoparticles.^[11]^”

The medicinal genus Vaccinium, belonging to the order Ericales and the family Ericaceae, comprises popular plants whose medicinal, nutritional, and industrial value is increasing every day. There are 100 species of this genus worldwide and they are considered rich sources of phenolic compounds and triterpenes, including flavonols, phenolic acids, and anthocyanins.^[12]^ These active phytochemicals display a strong potential for antioxidant activity and the ability to alleviate different chronic diseases.^[13]^ Among the polyphenols present in blueberries, particularly anthocyanins, have been reported to be strong reducing agents.^[14]^ This strong antioxidant capacity can be utilized in the reduction of metallic ions such as silver to form nanoparticles.^[15]^

In this work, we developed a methodology to obtain Ag@Au configuration in the form of nanostars with plasmonic properties through green synthesis, offering an environmentally friendly and safe alternative to traditional methods using plant extracts as reducing agents in the synthesis process. Subsequently, we characterized these nanoparticles to understand their structural and plasmonic properties. and evaluated their cellular internalization, and assessed their cytotoxicity through specific assays, ensuring a comprehensive understanding of their biological behaviour.

Our goal is to study their potential utility in cancer treatment in future research.

## 2 Results and Discussion

### 2.1 Antioxidant Capacity of *V. Corymbosum* Extract

The measurement of the antioxidant capacity of the *V. corymbosum* extract gives EC_50_ value 0.0794±0.0036 μg/ml which is a very small value compared to extracts from other plants such as *Thymus vulgaris* (12.10 μg/ml), *Pimpinella anisum* (18.12 μg/ml), *Mentha pulegium* (16.92 μg/ml), or *Stachys annua* (7.80 μg/ml)^[16]^ that are considered powerful antioxidants due to their phytochemical composition. The very high antioxidant capacity obtained for *V. corymbosum* may be due to the high bioactive concentration of our ethanolic extract. To obtain this, after ethanolic extraction, the extract was freeze‐dried, producing a high concentration of bioactive species since blueberries can have more than 80 % water. In the other reported cases the EC_50_ determinations were made on non‐lyophilized ethanolic extracts. In any case, the antioxidant capacity of the *V. corymbosum* is sufficient to make it suitable for the green synthesis of metal nanoparticles.

### 2.2 Structural Characterization and Nanoparticle Size

**Figure 1:**
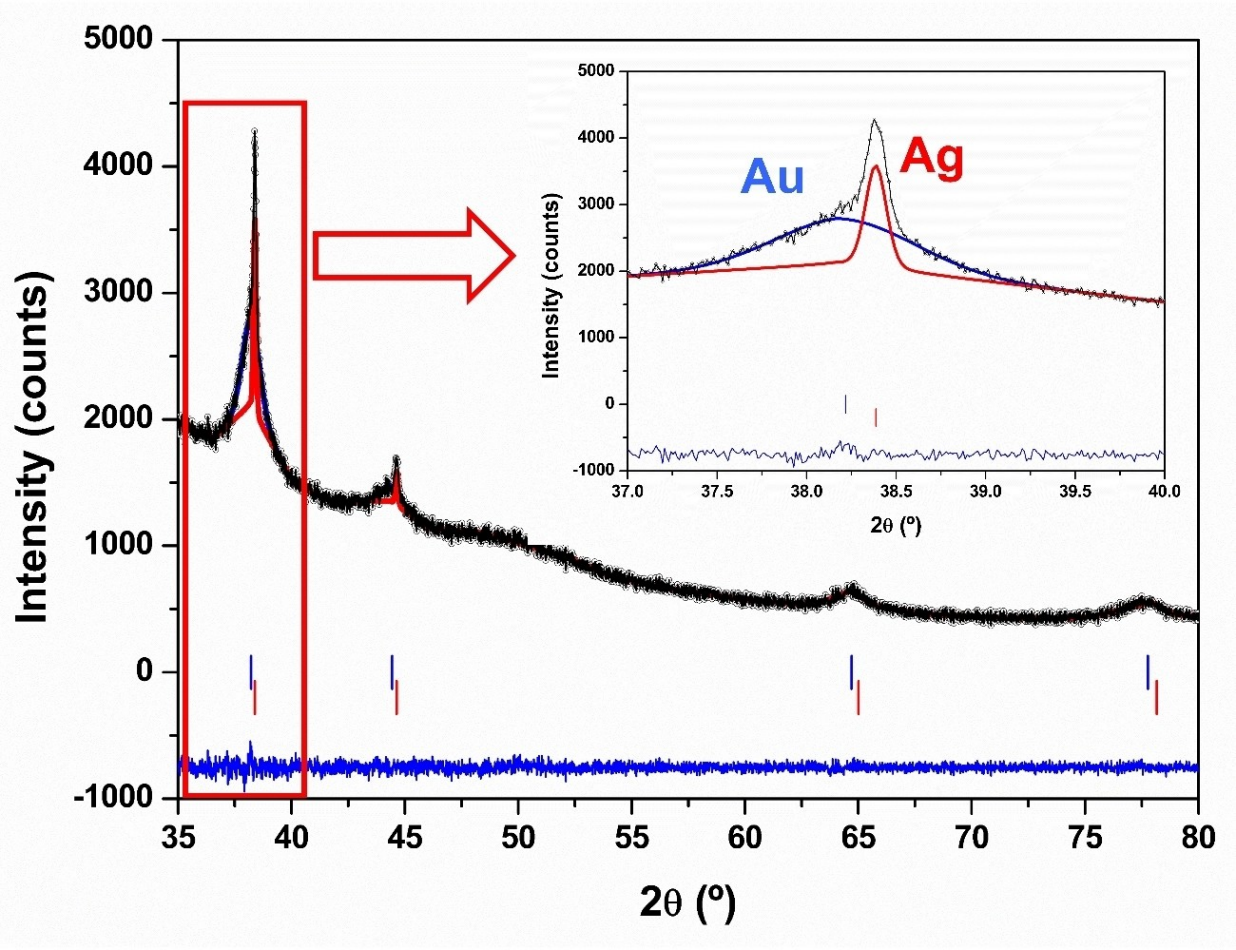
Fitting the X‐ray diffraction pattern of the Ag@Au sample to a structural model containing Ag and Au. The black points are the experimental pattern, the continuous black line the calculated one and the blue line at the bottom their difference. The red bars indicate the positions of the diffraction peaks of Ag and the blue ones those of Au phase. The inset shows a zoom of the area centred at 38.5°(2θ) where the most intense maxima of both metals appear.

Figure 1 shows the XRD pattern of the Ag@Au sample. Both metallic Au and Ag are observed. The maxima in the case of Au are wider indicating a smaller crystalline domain size than for Ag, whose maxima are narrower (inset of Figure 1).

In Ag@AuNPs the structure of both metals is the usual cubic compact with space group (S.G.) Fm3m. The lattice parameters are a=4.0512(6) Å and a=4.0801(5) Å, for Ag and Au, respectively. The microstructural information provided by the fitting of the XRD patter suggests that the nanoparticles consist of Ag areas of 76 nm diameter, and Au zones with a thickness of 10 nm. The exact distribution of those “areas” of different metals can only be determined by combining different techniques including HTREM. As discussed below, the most likely structure is a core‐shell like. Assuming this structure, the nanoparticles consist of a 76 nm core covered by 10 nm shell, thus, their total size is around 96 nm. The calculation of the nanoparticles size using the information on the domain sizes obtained from the XRD fitting, is a rough estimate that requires validation by direct observation by electron microscopy. SEM images of the nanoparticles have been taken, where a size of around 50 nm can be estimated (Figure SI1), in good agreement with the value obtained by HRTEM as explained below. This value is slightly lower than that estimated by XRD.

The particle D_H_ size obtained by DLS is 78 nm with a polydisperse index of 0.436. The former value is close to that determined by XRD (96(10) nm) whereas the latter suggests that the Ag@AuNPs are prone to aggregate, as observed in Figure SI1.

**Figure 2:**
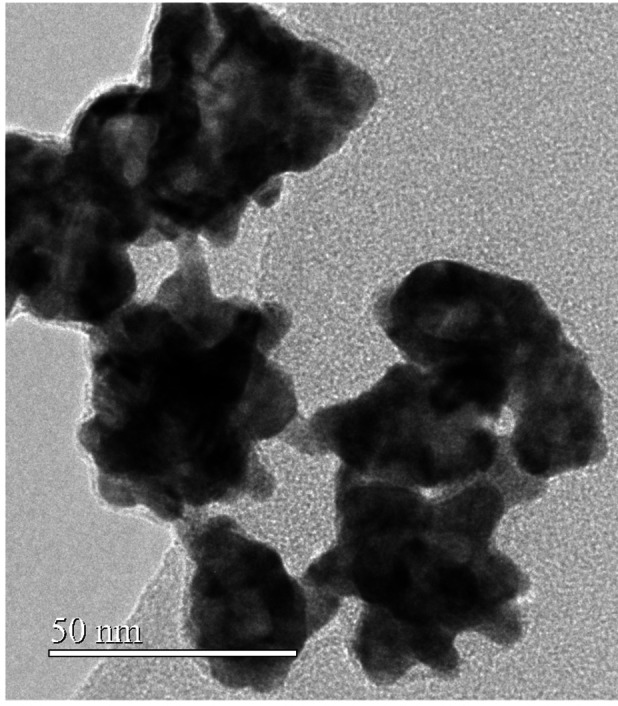
HRTEM images of Ag@AuNPs showing the nanostar shape.

**Figure 3:**
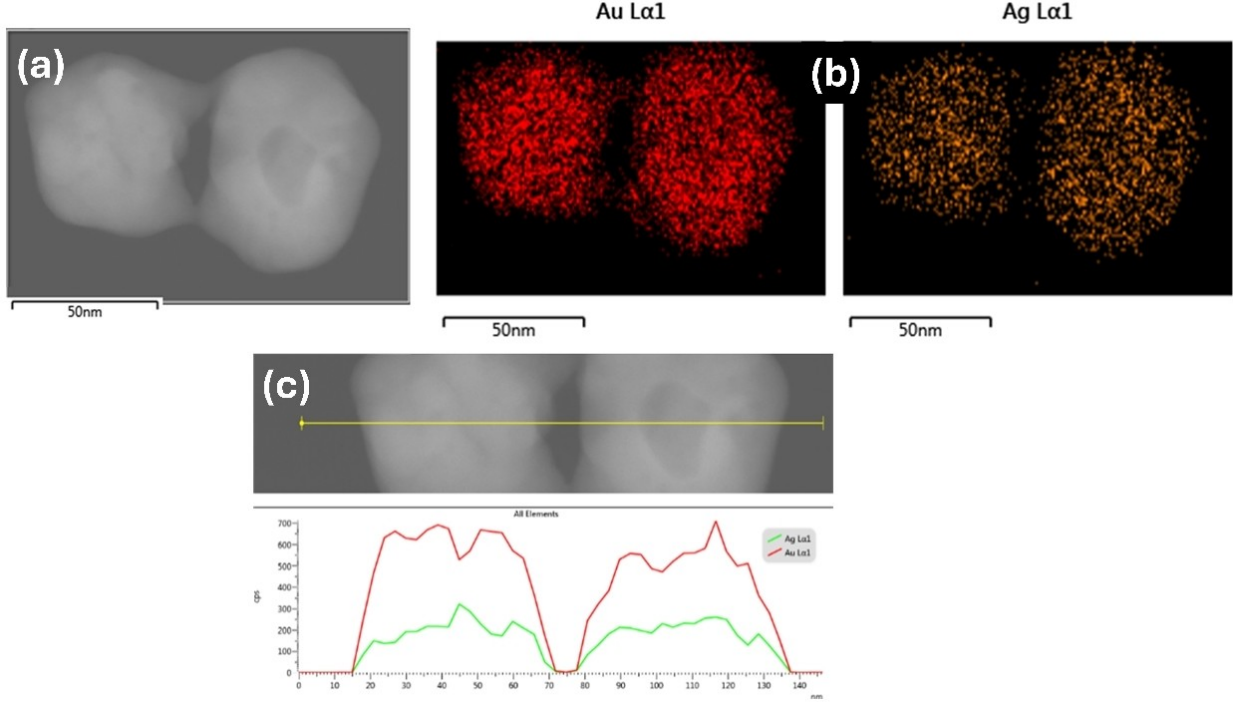
STEM (a) and EDS mapping (b) of a pair of Ag@AuNPs. (c) Scan line of Lα1 corresponding to Au and Ag.

Further characterization of Ag@AuNPs has been performed by HTREM. Conclusive and direct evidence about the size and shape of the nanoparticles are obtained by this technique; as Figure 2 and 3 show, the particles are nanostars with an average diameter around 50 nm. Among the different determinations of the nanoparticles size, this is the most reliable since is obtained by direct inspection, besides, it is confirmed their tendency to aggregate.

Figure 3(a) corresponds to a STEM image of two nanoparticles, on which the EDS analysis shown in Figure 3(b) was performed. This analysis demonstrates that Ag and Au are homogeneously distributed along the sample, all the nanoparticles contain both metals, being the composition of the nanoparticle similar. Figure 3(c) shows the scan line of Lα1 corresponding to Au and Ag. The present images do not provide direct evidence on the atomic distribution of the elements, i. e. whether the particles are formed by an Ag−Au alloy, or they present a core‐shell structure. However, a Janus‐like structure and the formation of segregated Ag and Au nanoparticles can be ruled out. From the XRD pattern the formation of an alloy can be also discarded since the peaks of both metals are observed. Therefore, the most likely structure is a core‐shell one; radiation absorption measurements support this model since only one absorption maximum is observed^[17]^ (see below).

**Figure 4:**
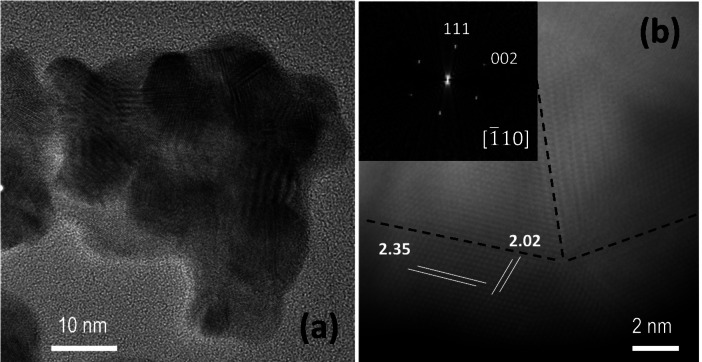
(a) HRTEM of an Ag@AuNP and (b) HRTEM image of Ag@AuNP taken along [$\bar{1}$ 10], in the inset the FFT of the image is shown.

Figure 4(a) depicts the HRTEM image of an aggregate of NPs, whereas Figure 4(b) shows the image of one of those NPs and the corresponding FFT in the inset. The NPs are crystalline presenting the characteristics d‐spacing, (111) and (002), of the cubic structure of the metals. The splitting of the spots in the inset of Figure 4(b) is due to the presence of different domains clearly observed in the image. It is not possible to assess this microstructure to the Au−Ag core‐shell particle structure or simply to the intergrowth of domains of a given metal.

### 2.3 Elemental Analysis and Concentration of Nanoparticles Suspensions

To estimate the nanoparticles concentration of the suspensions, elemental analysis by ICP were performed. The contents of Au and Ag (in μg/ml) are: 6.7(4) and 7.6(6), respectively, which correspond to Au/Ag weight ratio 47/53.

To calculate the concentration of NPs, the size of 50 nm (given HRTEM) was used as well as the corresponding metal densities in g.cm^−3^ (Ag: 10.49, Au: 19.3). Assuming a spherical core‐shell shape, an estimation of the diameter of the Ag‐core (d_Ag_), the thickness of the outer Au layer (L_Au_) and the concentration of the suspensions (C_NP_) are calculated to be: d_Ag_=42 nm, L_Au_=4 nm, and C_NP_=1.87 10^10^ NPs/ml. The wo former values are different but comparable to those obtained by XRD.

### 2.4 Radiation Absorption and Hyperthermia

**Figure 5:**
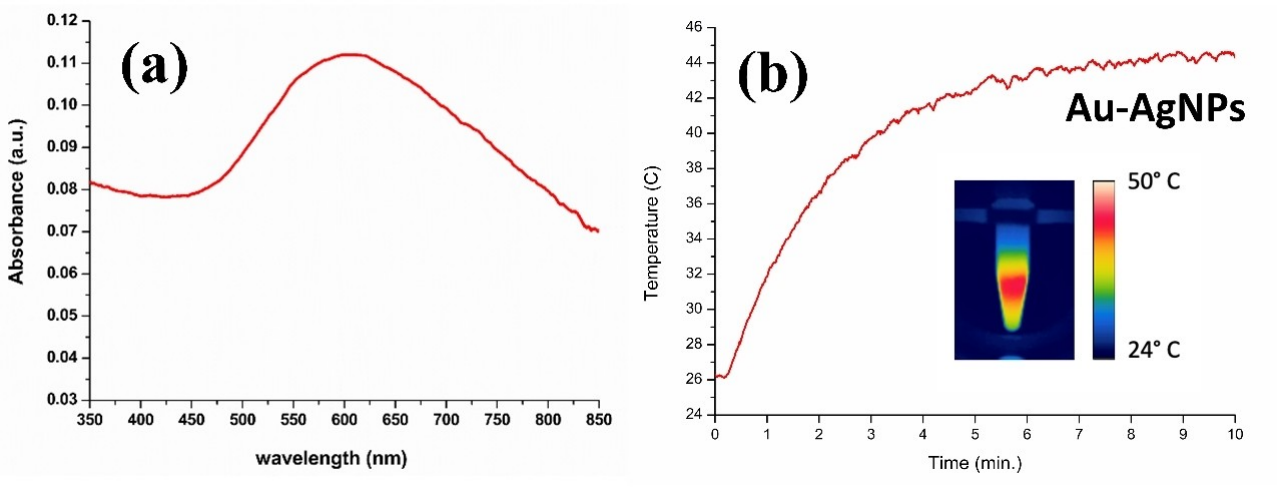
(a) UV–vis–NIR absorption spectrum of Ag@AuNPs. (b) Graphic representation of temperature (Celcius) versus time (min) of the Ag@AuNPs samples irradiated with a 680 nm laser light source and capture of the final thermographic image.

The absorption band corresponding to the localized resonance surface plasmon (LSPR) appears in Ag and Au at around 420 and 520 nm, respectively, depending on the nanoparticle shape and size characteristics and dielectric properties of the surrounding medium.^[18]^ Figure 5(a) depicts the absorption spectrum of the Ag@AuNPs, showing a broad and prominent band between 600–700 nm. The extinction coefficient estimated at 610 nm, at which an absorbance of 0.11 is measured and considering the above NPs concentration with the experimental optical path, results to be ϵ_610_=3.30 10^10^ M^−1^ cm^−1^; which is a large value similar to those previously reported for similar nanoparticles.^[5a]^ As result of the characterization techniques discussed above, the present NPs are composed of a star‐shaped Au‐coating onto a silver core. In case of a bimetallic configuration (Janus‐like structure), two differentiated bands corresponding to the LSPR of both metals should be observed in the absorption spectrum.^[19]^

According to the absorption spectrum on Figure 5(a), a photothermal effect should be observed under laser excitation of wavelength λ=680 nm. Figure 5(b) shows a graphic representation of temperature (Celsius) against time (min) the sample was exposed to the light source. The gold‐coated silver nano stars reach a temperature of 44.3 °C after 10 minutes of exposure. To highlight the evolution of temperature over time, a video is included in the supplementary material. For comparison also included in the supplementary material is the temperature variation over time of AuNPs **(**Figure SI2**)** exhibiting an LSPR around 540 nm, showing heating after 10 minutes using a 680 nm laser source, reaching 37.9 °C.

Worth to note, to be use in photoexcited hyperthermia therapies, the optimal temperature that metallic NPs must reach ranges from 42–46 °C.^[20]^ Our results suggest that the present Ag@AuNPs deserve some biological studies, to assess their potential use for hyperthermia therapies.

### Internalization of Ag@AuNPs by Macrophages, Fibroblasts and Cancer Cells

**Figure 6:**
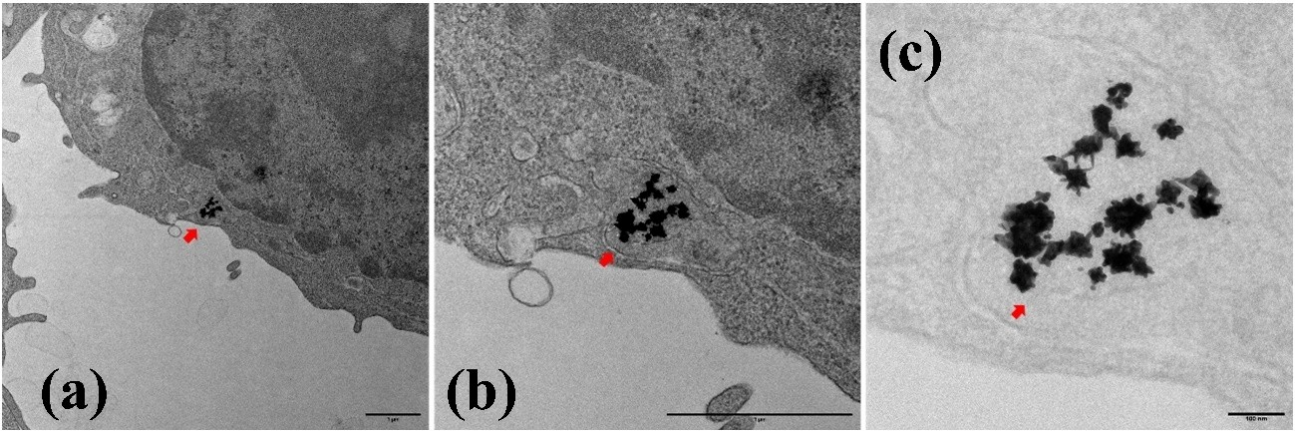
Uptake of Ag@AuNPs by RAW macrophage. Subpanel (a) represents the TEM image of macrophage treated with nanoparticles at magnification x6k, scale bar 1 μm, (b) and (c), are the higher magnification images of (a). (b) x20k, scale bar 1 μm and (c) x60k, scale bar 100 nm. The cells treated with Ag@Au NPs (0.1 μg/ml of Au) evidenced presence of these within the cellular compartment, most likely in the endosomal system (arrows).

**Figure 7:**
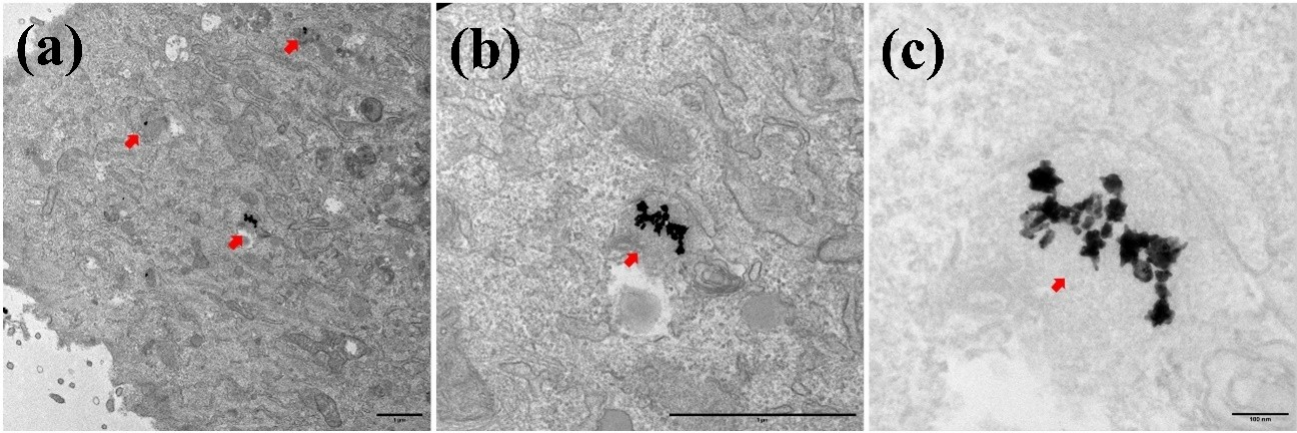
Uptake of Ag@AuNPs by Fibroblasts HS27. Subpanel (a) represents the TEM image of fibroblasts treated with nanoparticles at magnification x5k, scale bar 1 μm, (b) and (c), are the higher magnification images of (a). (b) x20k, scale bar 1 μm and (c) x60k, scale bar 100 nm. The cells treated with Ag@AuNPs *(*0.1 μg/ml of Au*)* evidenced presence of these within the cellular compartment, most likely in the endosomal system (arrows).

**Figure 8:**
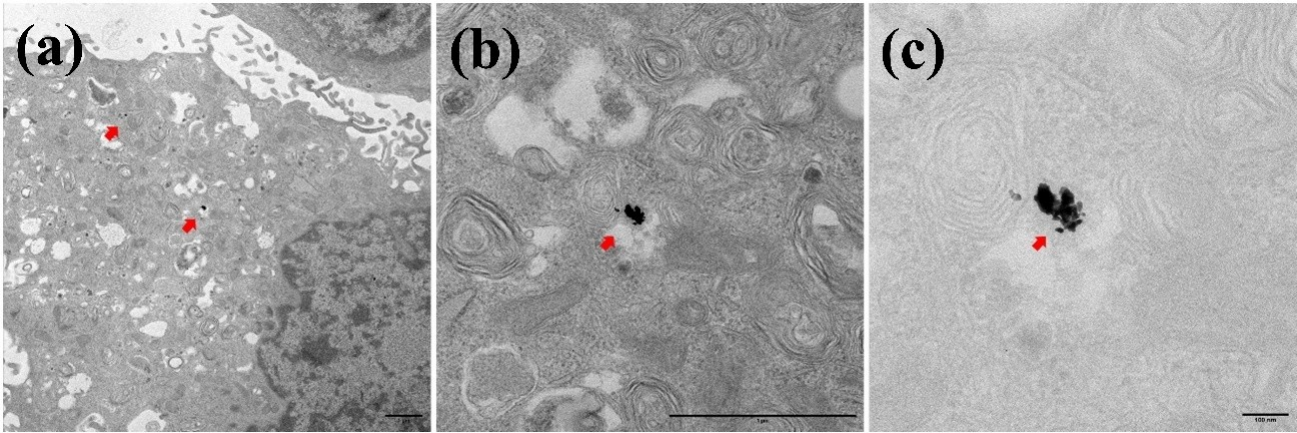
Uptake of Ag@AuNPs by Cancer cells 4T1. Subpanel (a) represents the TEM image of fibroblasts treated with nanoparticles at magnification x4k, scale bar 1 μm, (b) and (c), are the higher magnification images of (a). (b) x20k, scale bar 1 μm and (c) x60k, scale bar 100 nm. The cells treated with Ag@AuNPs *(*0.1 μg/ml of Au*)* evidenced presence of these within the cellular compartment (arrows).

The results demonstrate that all cell lines studied in this work uptake Ag@AuNPs aggregates (Figures 6, 7 and 8). TEM images showed, in all three cases, the presence of Ag@AuNPs inside the cells. In macrophages (Figure 6) and fibroblasts **(**Figure 7 **)**, the NPs are located intracellularly in large aggregates, surrounded by membranes corresponding to endosomes. In 4T1 cells (Figure 8), the NPs are also found forming aggregates in the cytoplasm; however, they do not appear to be within membrane‐surrounded compartments.

The successful incorporation of Ag@AuNPs into diverse cell types such as macrophages, fibroblasts, and cancer cells represent a notable advantage for their broad use in biomedicine, as well as their potential for personalized medicine and the treatment of complex diseases. These NPs can be designed to interact specifically with cellular components, either by modifying their surface with specific ligands or by manipulating their plasmonic properties. However, the passage of nanoparticles through the cell plasma membrane is influenced by ligand‐receptor interactions, surface charge, hydrophobicity, size, and shape, determining different absorption mechanisms depending on the cell type.^[21]^ In cellular therapy, macrophages and fibroblasts can serve as delivery vectors to transport NPs loaded with drugs or therapeutic agents to specific sites in the body.^[22]^ Their incorporation into cancer cells offers the possibility of using them in early cancer detection, as well as in the development of therapies targeted specifically at tumour cells, leveraging plasmonic properties to induce selective cell death through photothermal therapy or controlled drug release. The promising advancements in nanoparticle research, exemplified by ongoing clinical trials such as AuroLase for tumour ablation, underscore the significance and success of these investigations in current biomedical practices.^[23]^

**Figure 9:**
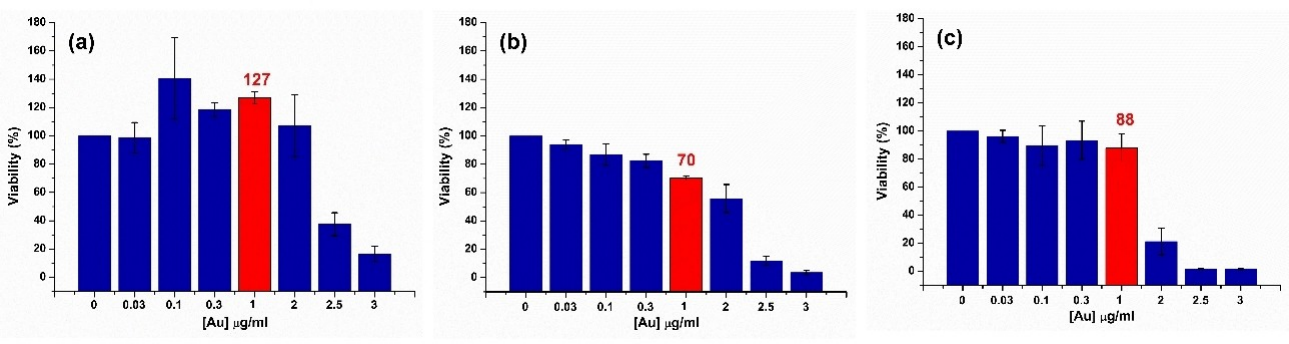
Evaluation of the cytotoxic effect of Ag@AuNPs on fibroblasts (a), macrophages (b), and cancer cells (c). Cytotoxicity was assessed using the Alamar Blue assay, measuring fluorescence in cells treated with nanoparticles at concentrations ranging from 0.03–3 μg/ml of Au. Controls, including untreated cells, were included for comparison. Results are expressed as the mean±SD of three independent experiments (n=3). This analysis provides insights into the biocompatibility of the nanoparticles across the three cell lines employed in this study.

Figure 9 depicts the results of the viability tests performed on each of the cell lines in this study. All the cells are viable when the cytotoxicity was analysed at the [Au] concentration (0.1 μg/ml) used in the internalization processes.

At a [Au] concentration of 1 μg/ml, the cell viability of most lines studied remained unchanged, except for macrophages (Figure 9(b)), where 30 % cell death was observed. This result aligns with expectations, as macrophages possess higher phagocytic capacity.

When the concentration was increased to 2 μg/ml, a slight additional decrease in macrophage viability was observed, reaching 40 % cell death, representing a 10 % increase compared to the value obtained at 1 μg/ml. In contrast, fibroblasts showed no significant changes (Figure 9(a)) in viability at this concentration. On the other hand, tumour cells exhibited marked sensitivity (Figure 9(c)), with only 20 % cell viability at 2 μg/ml of [Au].

The results obtained clearly show a difference in the response to Ag@AuNPs between tumour cells and non‐tumour cells, such as fibroblasts and macrophages. At a concentration of 2 μg/ml of Au, tumour cells exhibited a significant reduction in viability (80 % cell death), while fibroblasts showed no significant effects, suggesting a high selectivity of the nanoparticles for tumour cells. This finding is relevant for the development of targeted therapies, as Ag@AuNPs could be used to specifically target cancer cells, minimizing damage to surrounding healthy tissues.

On the other hand, macrophages displayed an intermediate response, with 40 % cell death at 2 μg/ml of Au, reflecting their superior phagocytic capacity. However, cell viability in macrophages remained considerably higher than in tumour cells, supporting the possibility of using Ag@AuNPs in therapies with a good safety profile.

The ability of Ag@AuNPs to induce selective cytotoxic effects in tumour cells, combined with their potential hyperthermic effect, which could enhance cancer cell destruction, underscores their potential as a therapeutic tool in cancer treatment. These results open new perspectives for the design of more specific and less invasive anticancer therapies.

## 3 Conclusions

An eco‐friendly method was developed to synthesize gold‐coated silver nanoparticles (Ag@AuNPs) in a star‐like shape using *Vaccinium corymbosum* extract as a reducing agent. The nanoparticles exhibit a core‐shell structure with a 40 nm silver core and a 5 nm gold shell, as confirmed by XRD, DLS, SEM, and HRTEM/STEM.

The absorption of these NPs in the visible region makes them suitability for photothermal applications: when excited with a laser at 680 nm for 10 minutes the temperature rises to 44.3 °C which is optimal for hyperthermia therapy.

Ag@AuNPs were internalized by macrophages, fibroblasts, and 4T1 cancer cells, with intracellular aggregates found in endosomal membranes (macrophages and fibroblasts) or freely in the cytoplasm (4T1 cells). These nanoparticles exhibit selective, concentration‐dependent cytotoxicity, effectively targeting tumour cells while sparing healthy cells like fibroblasts. Their intrinsic toxicity, independent of hyperthermia, highlights their potential as a safe and promising therapeutic tool, though macrophage sensitivity should be considered in future designs.

This work demonstrates a sustainable approach to synthesizing functional nanoparticles for nanomedicine, aligning with green chemistry and biocompatibility principles.

## Experimental Section

### Synthesis

Ag@AuNPs are synthesized at room temperature using an ethanolic solution of *Vaccinium corymbosum* L. extract with a concentration of 400 mg/L as reducing agent. 1 ml of the blueberry extract solution is added to 20 ml of a 1 mM AgNO₃ (Sigma Aldrich ≥99 %) aqueous solution and stirred for 10 minutes, at which point the solution turns pink. Next, the mixture is placed in an ultrasonic bath for 15 minutes, and 500 mM NaOH is added drop by drop until a pH of 10 is reached. At this point, the colour changes from pink to brown. The mixture is centrifuged at 2400 rpm for 30 minutes, the supernatant is discarded, and a precipitate is obtained, which is redispersed in 10 ml of ultrapure water and placed in an ultrasonic bath for 10 minutes.

An aliquot of 5 ml of the previously obtained suspension is taken, and 0.2 ml of 100 mM ascorbic acid (Sigma Aldrich) and 2 ml of 2.9 mM AuCl₃ (Sigma Aldrich 99 %) are added. The solution then changes colour to blue. This mixture is shaken vigorously for 20 minutes and then centrifuged at 2400 rpm for another 20 minutes. A blue precipitate is obtained, which is redispersed in 10 ml of ultrapure water (Figure SI3).

Gold nanoparticles, AuNPs, have been synthesized for comparison purposes following the synthesis procedure provided in the supplementary material (Figure SI3).

### Determination of the Antioxidant Capacity of Vaccinium Corymbosum Extract

The extract of *V. corymbosum* was provided by the biotechnology company Exxentia (currently part of the Kerry Food Group P.L.C.). The radical scavenging activity of *V. corymbosum* extract against 1,1‐diphenil‐2‐pricrilhidrazil (DPPH) free radical (antioxidant potential) was measured using the method described in reference.^[24]^ The antioxidant potential EC_50_ (quantity of blueberry extract needed to reduce to 50 % the initial concentration of free radicals of DPPH solution) was determined by a curve dilution made with different blueberry extract concentrations mixed with 0.1 mM DPPH in methanol 80 %. The absorbance at 517 nm was measured after standing in the dark for 30 min. Results were expressed as percentage of inhibition=[(Abs0–Abs1)/(Abs0)]×100, where Abs0 was the absorbance of the control (DPPH without extract) and Abs1 was the absorbance in presence of the extract.^[25]^ The blank was made with methanol. From its definition above, the lower values EC_50_ the higher antioxidant (reducing) potential.

### Characterization Techniques of NPs

The concentrations of Au and Ag in Ag@AuNPs are determined with inductively coupled plasma optical emission spectroscopy (ICP‐OES) in both acidic and aqueous medium. The concentration of metallic NPs was estimated from the elemental concentration, the size of the nanoparticles and the density of silver and gold (10.49 and 19.3 g/cm^3^, respectively) assuming the nanoparticles to be spheres.

Sample purity, crystal structure and domain size, were determined from powder X‐ray diffraction (PXRD) data collected on a Bruker D8 high‐resolution diffractometer equipped with a LynxEye fast detector using monochromatic Cu Kα1 (λ=1.5406 Å) radiation obtained with a germanium primary monochromator. The angular range, step size, and counting times were selected to ensure the required data quality and resolution for structural refinement. Diffraction data were analysed using the Fullprof software.^[26]^ The peak shape was described by a pseudo‐Voigt function, and the background level was fitted by linear interpolation.

To estimate the domain size, the instrumental resolution function (the contribution of the instrument to the peak widths) was determined using a NIST LaB6 standard, and the simultaneous refinement of the phases' structural and microstructural features (domain size and stress) was performed using the FullProf software.^[26]^

For the dynamic light scattering (DLS) measurements to obtain the hydrodynamic diameter (DH), a Zetasizer Nano (Malvern Instruments) was used. Measurements are made in a suspension of the sample in water at pH 7 in a standard polystyrene cuvette; the temperature is set to 24.9 °C, each measurement was recorded 60 seconds to reach a signal of 206.0 kcps and the refractive index applied is that of gold (0.2).

The spectra were made with the samples suspended in ultrapure water. A dual‐beam UV‐3100 UV–VIS–NIR Recording Spectrophotometer (Shimadzu) was used in a wavelength range between 200 and 1000 nm using quartz cuvettes with a pitch of 1 mm. This spectrophotometer is capable of measuring in three different spectral ranges UV/VIS/NIR.

To obtain the scanning electron microscopy images (SEM), a Thermo Fisher Scientific Prisma E with xT Microscope Control v16.2.2 software was used. Few drops of the NPs suspensions in water were dropped on the holder sample, after dried, the sample was observed working in Secondary Electrons mode at high vacuum, at 30 kV and 24 pA.

Ag@AuNPs were further characterized by High Resolution Transmission Electron Microscopy (HRTEM) using a JEOL JEM GRAND ARM 300cF equipped with a Cs Corrector (ETA‐JEOL). Local composition was analysed by EDS analysis SDD CENTURIO Detector.

The equipment to measure the photoexcited hyperthermia of suspensions of the nanoparticles in water, incorporates a 680 nm continuous laser with a power of 0.3 W (Laser Components), selected based on the maximum of plasmon of the sample. In addition, the equipment includes a thermal infrared (IR) imaging camera that provides an additional tool for the detection and monitoring of IR radiation and the temperature distribution in the sample and over time. The photoexcited hyperthermia treatment, was performed during 10 min, during this time the suspension temperature is monitored by IR emission with an IR camera (FLIR).

### Cells

Mouse peritoneal macrophage cells, RAW264.7 (ATCC) and breast human cancer cell derived from mammary gland tissue of BALB/c strain mice, 4T1 (ATCC) were cultured in RPMI medium with 10 % heat‐inactivated fetal bovine serum (FBS) and 1 % penicillin streptomycin. Human fibroblasts, Hs27 (ATCC), were cultured in Dulbecco's Modified Eagle Medium (DMEM) medium with 10 % FBS and 1 % penicillin streptomycin. Cell lines were maintained under standard conditions (5 % CO2, 37 °C). All reagents used in culture were purchased from (Thermo Fisher Scientific).

### Uptake of o Ag−AuNPs by Cells

Preconfluent cell cultures (2×10 6 cells) were treated with gold concentrations of 0.1 and 2.5 μg/ml (corresponding to C_NP_=2.8 10^8^ and 7.0 10^9^ NPs/ml, respectively) for 48 hours at 37 °C and 5 % CO_2_. At the end of the incubation period, the supernatant was removed, and fixation was carried out for subsequent analysis by transmission electron microscopy (TEM). For TEM, cells (Mock and incubated with Ag@AuNPs) were fixed with 4 % PFA and 2 % glutaraldehyde in 0.1 M phosphate buffer (PB; pH 7.4) for 90 min at room temperature. Postfixation was carried out with 1 % OsO_4_ and 0,8 % K_3_[Fe(CN)_6_] in water at 4 °C for 1 h. Samples were dehydrated with ethanol and in situ flat embedded in Taab 812 epoxy resin (Taab Laboratories) according to standard procedures. After polymerization, resin sheets containing the cell monolayers were detached from the substrate and mounted onto resin blocks to obtain orthogonal (from the bottom to the top of the cell) 70–80 nm ultrathin sections. The sections were deposited onto slot grids and stained with uranyl acetate and lead citrate. Grids were examined at 100 kV in a JEOL JEM 1400 Flash electron microscope, and all images were recorded with a TemCam‐F416 (4K4K) digital camera from Tietz Video and Image Processing Systems (TVIPS; Martinsried, Germany).

### Cytotoxicity Studies

The cytotoxicity of Ag@Au nanoparticles (NPs) was evaluated on macrophages (Raw 264.7), human fibroblasts (Hs27), and cancer cells (4T1) using a colorimetric assay with alamarBlue® reagent (Invitrogen, USA). Cells were harvested by centrifugation at 300 g for 10 minutes and washed three times with PBS. Subsequently, cells were seeded into 96‐well culture plates (Costar Corning, USA) at a density of 10^5^ cells/well. Experimental groups included untreated controls (negative control) and treatments with suspension of Ag@Au NPs of concentrations, using Au concentration as reference, ranging from 0.03 μg Au/ml–3Au μg/ml; which corresponds to 8.4 10^7^ and 8.4 10^9^ NPs/ml, respectively. All samples were incubated at 37 °C for 72 hours.

After the incubation period, alamarBlue® reagent was added to each well at 10 % of the total medium volume. Plates were then incubated for an additional 6 hours at 28 °C. Fluorescence was measured using a Varioskan™ LUX multimode microplate reader with excitation and emission wavelengths set to 530 nm and 590 nm, respectively.

4

## Conflict of Interests

The authors declare no conflict of interest.

## Supporting information

As a service to our authors and readers, this journal provides supporting information supplied by the authors. Such materials are peer reviewed and may be re‐organized for online delivery, but are not copy‐edited or typeset. Technical support issues arising from supporting information (other than missing files) should be addressed to the authors.

Supporting Information

Supporting Information

Supporting Information

## Data Availability

The data that support the findings of this study are available from the corresponding author upon reasonable request.
