# Supplementary material for: Hyperthermic Core‐Shell Silver‐Gold Nanoparticles: Green Synthesis and Adsorption‐Uptake by Macrophages, Fibroblasts and Cancer Cells
Source: ChemistryOpen. 2025 Feb 19;14(3):e202400459. doi: 10.1002/open.202400459 (PMC12128158; doi:10.1002/open.202400459)
Supplement: Supplementary file 1 — Supporting Information [file OPEN-14-e202400459-s002.pdf]

ChemistryOpen

Supporting Information

Hyperthermic Core-Shell Silver-Gold Nanoparticles: Green Synthesis and Adsorption-Uptake by Macrophages, Fibroblasts and Cancer Cells

E. Valdivieso, M. Zabala, A. Muñoz Noval, R. López-Méndez, N. Carmona, A. Espinosa, F. J. García García, K. Boulahya, J. A. Lucas, L. Biancotto, U. Amador, M. T. Azcondo,* and C. Hurtado-Marcos

Supporting Information

Hyperthermic Core-Shell, Silver-Gold Nanoparticles: Green Synthesis and adsorption-uptake by macrophages, fibroblast and cancer cells

E. Valdivieso[1], M. Zabala[2], A. Muñoz Noval[3,4], R. López-Méndez[4], N. Carmona,[3] A. Espinosa,[5] F.J. García García,[6] K. Boulahya,[7] J. A. Lucas [1], L. Biancotto,[2] U. Amador,[2] M. T. Azcondo,[2,] C. Hurtado-Marcos[1]*

[1] Plant Physiology, Pharmaceutical and Health Sciences Department, Faculty of Pharmacy, Universidad San Pablo-CEU, CEU-Universities, 28668 Boadilla del Monte, Spain

[2] Universidad San Pablo-CEU, CEU Universities, Facultad de Farmacia, Departamento de Química y Bioquímica, Urbanización Montepríncipe, Boadilla del Monte, E-28668, Madrid, Spain

[3] Departamento de Física de Materiales, Facultad de Físicas, Universidad Complutense, E-28040, Madrid, Spain.

[4] IMDEA Nanociencia c/ Faraday, 9, Madrid 28049, Spain.

[5] Instituto de Ciencia de Materiales de Madrid, Consejo Superior de Investigaciones Científicas, calle Sor Juana Inés de la Cruz 3, 28049-Madrid, Spain

[6] ICTS-Centro Nacional de Microscopía Electrónica F. CC. Químicas, UCM Av. Complutense S/N 28040-Madrid, Spain

[7] Departamento de Química Inorgánica I, Facultad de Ciencias Químicas, Universidad Complutense, 28040, Madrid, Spain

*Corresponding author: *azcondo@ceu.es*

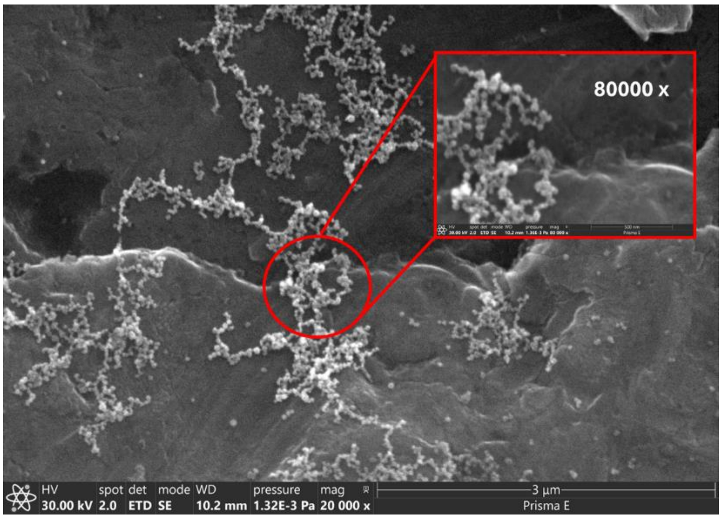

Figure SI1. SEM image of the Ag@Au nanoparticles showing the formation of collar-like aggregates. In the inset, NPs are shown at a magnification of 80000x.

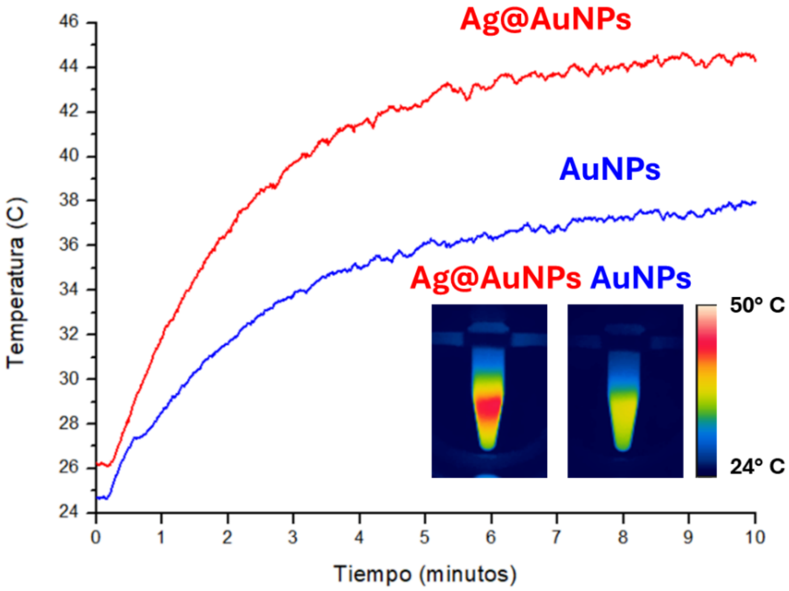

Figure SI2. Graphical representation of the temperature (Celsius) evolution over time (minutes) of Ag@AuNPs and, for comparison, of AuNPs. The laser used for heating is 680nm.

Synthesis of AuNPs

To synthesize AuNPs, take 5 mL of 2.9 mM $AuCl_3$ solution, 15 mL of miliQ water and 2 mL of an ethanolic solution blueberry extract of concentration 400 mg/L is used. Stir this mixture vigorously at RT for 20 minutes, then add 500 mM NaOH is added drop by drop until a pH of 10 is reached. Then centrifuge at 2400 rpm for 30 minutes. The resulting purple precipitate is shown in (**Figure SI3**)

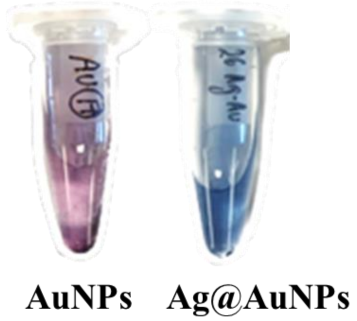

Figure SI3. A purple suspension of the AuNPs (left) and a blue suspension of Ag@AuNPs (right).
